# Lipid Nanoparticles for Nucleic Acid Delivery to Endothelial Cells

**DOI:** 10.1007/s11095-023-03471-7

**Published:** 2023-02-03

**Authors:** Gary W. Liu, Edward B. Guzman, Nandita Menon, Robert S. Langer

**Affiliations:** 1 Koch Institute for Integrative Cancer Research, Massachusetts Institute of Technology, Cambridge, MA 02139 USA; 2 Department of Biology, Massachusetts Institute of Technology, Cambridge, MA 02139 USA; 3 School of Engineering and Applied Sciences, Harvard University, Cambridge, MA 02138 USA; 4 Present Address: Strand Therapeutics, MA 02215 Boston, USA

**Keywords:** endothelial cells, gene delivery, lipid nanoparticle, nanoparticles, nucleic acids

## Abstract

Endothelial cells play critical roles in circulatory homeostasis and are also the gateway to the major organs of the body. Dysfunction, injury, and gene expression profiles of these cells can cause, or are caused by, prevalent chronic diseases such as diabetes, cardiovascular disease, and cancer. Modulation of gene expression within endothelial cells could therefore be therapeutically strategic in treating longstanding disease challenges. Lipid nanoparticles (LNP) have emerged as potent, scalable, and tunable carrier systems for delivering nucleic acids, making them attractive vehicles for gene delivery to endothelial cells. Here, we discuss the functions of endothelial cells and highlight some receptors that are upregulated during health and disease. Examples and applications of DNA, mRNA, circRNA, saRNA, siRNA, shRNA, miRNA, and ASO delivery to endothelial cells and their targets are reviewed, as well as LNP composition and morphology, formulation strategies, target proteins, and biomechanical factors that modulate endothelial cell targeting. Finally, we discuss FDA-approved LNPs as well as LNPs that have been tested in clinical trials and their challenges, and provide some perspectives as to how to surmount those challenges.

## Endothelial Cell Physiology in Health and Disease

**Fig. 1:**
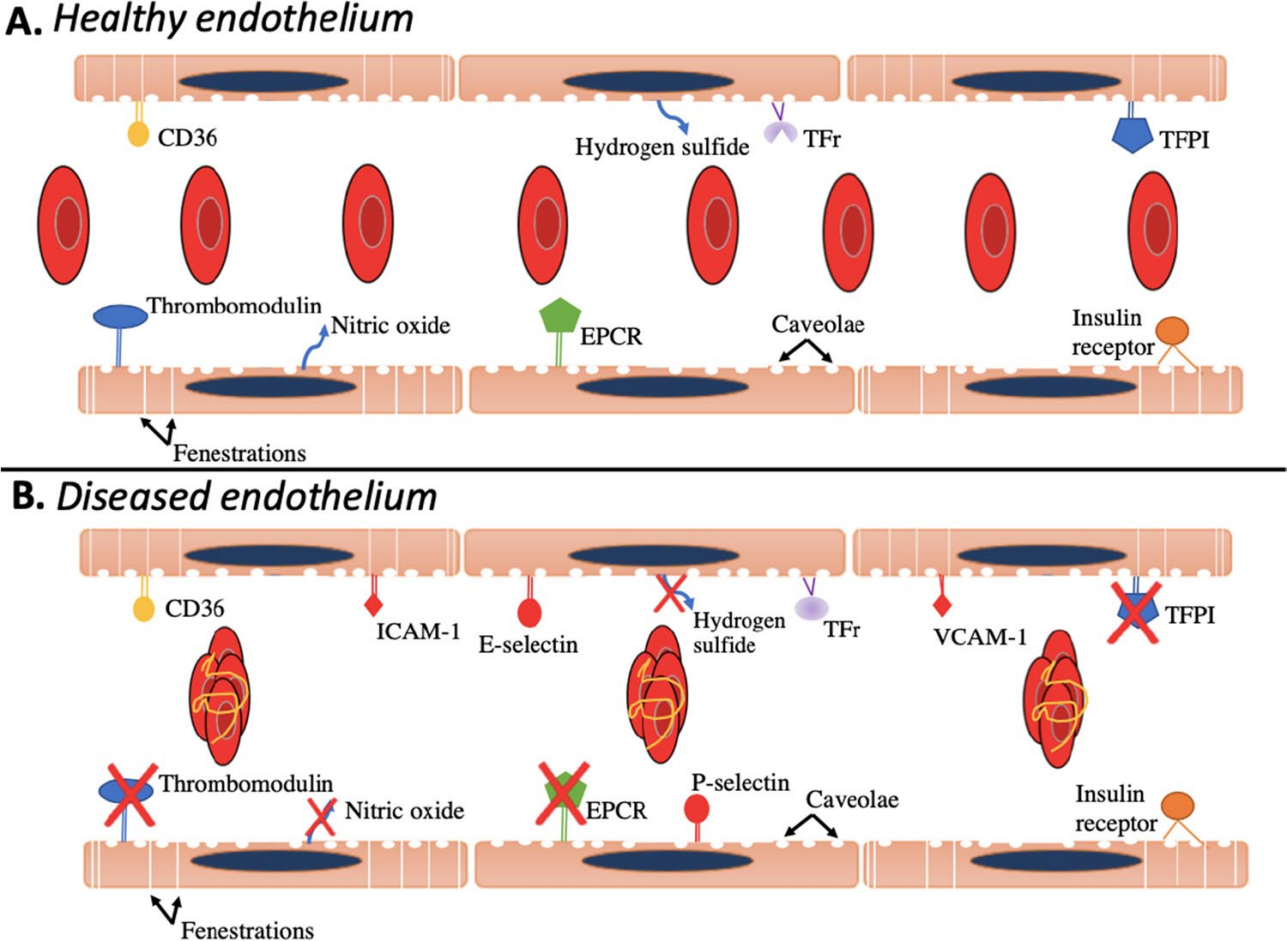
Endothelial cells in healthy and diseased environments. (**A**) Endothelial cells line the inner surface of blood vessels, controlling blood clotting, vessel size, immune function, and the passage of cells or molecules out of the circulation. By producing anticoagulant proteins such as thrombomodulin, TFPI, and EPCR, endothelial cells prevent clot formation inside blood vessels to enable proper oxygen and nutrient delivery to vascularized tissues in the body [3–6]. Endothelial cells also sense blood fluidity and produce gasses such as nitric oxide and hydrogen sulfide to regulate vascular flow [12, 19, 51]. Additionally, because endothelial cells are in direct contact with numerous blood components such as protein, sugars, and lipids, endothelial cells serve as a gateway for molecules to travel in-and-out of the circulation. CD36, transferrin, and insulin receptors along with caveolae on endothelial cells allow the passage of selective molecules out of the circulation, while fenestrations on endothelial cells enable the passage of low molecular weight solutes across the endothelium [22–28]. (**B**) When endothelial cells become dysfunctional, numerous physiological functions become altered, resulting in cardiovascular dysfunction that could lead to serious medical complications. For example, decreased production of anticoagulant proteins by endothelial cells could promote blood clots to form inside of blood vessels, altering blood fluidity and potentially inducing thrombosis [46, 52–54]. Decreased production of nitric oxide or hydrogen sulfide leads to inflammation and increases in blood pressure, conditions that could lead to atherosclerosis if they become chronic [12, 19, 55–57]. Systemic overexpression of adhesion molecules associated with inflammation, including P-selectin, E-selectin, ICAM-1, and VCAM-1, on endothelial cells can cause cardiac dysfunction [58–61].

Endothelial cells line the inner surface of blood vessels, with biological functions that are essential in maintaining a normal physiology. By controlling blood clotting, vessel size, and immune function, endothelial cells facilitate blood fluidity, oxygen distribution, cell transport, and nutrient supply to any tissue that is vascularized. This is primarily because endothelial cells are constantly producing anticoagulant proteins that prevent clotting inside vascular beds, which enables hemostasis and produces the appropriate blood flow and pressure needed to supply oxygen, nutrients, and cells to tissues [1–5]. Thrombomodulin, TFPI, EPCR, and heparin-like proteoglycans are examples of the major anticoagulants produced by endothelial cells whose primary function are to prevent platelet aggregation and fibrin formation inside blood vessels [3–7] (Fig. 1A). Endothelial cells also play critical roles during immune responses [8]. When an infection or injury occurs, endothelial cells release vasoactive mediators that locally increase the diameter of blood vessels to facilitate the passage of immune cells, and express adhesion molecules to allow leukocyte extravasation [9]. PGI2, nitric oxide (NO), and hydrogen sulfide are examples of vasodilators released by endothelial cells that increase vessel size by relaxing smooth muscle cells, while selectins and intercellular adhesion molecules (ICAMs) are examples of surface proteins that facilitate extravasation of immune cells out of the blood circulation and into the surrounding tissue [9–21] (Fig. 1A). Because endothelial cells are in direct contact with numerous blood components such as protein, sugars, and lipids, endothelial cells also serve as a gateway for molecules to travel out of the circulation. By expressing receptors that activate transcytosis, such as CD36, transferrin receptors (TFr), and insulin receptors, or by producing fenestrations along selective blood vessels, endothelial cells enable the selective transport of molecules from the circulation into the surrounding tissue [22–26]. For example, caveolae are membrane invaginations predominantly abundant on the surface of endothelial cells that enable the transport of a wide range of molecules across the endothelium via active transcytosis, while endothelial cells from specific vascular beds contain fenestrations that enable the passive transport of nanoscale molecules out of the circulation (Fig. 1A) [27, 28]. The liver and kidneys are organs known for having fenestrated endothelial cells, while the brain is characterized for its non-fenestrated and tightly packed endothelial cells [28–33].

Given the important and multiple biological functions of endothelial cells, coupled with their wide spread distribution in the body, endothelial cells contribute to a wide range of diseases and life-threating conditions [34–45]. Accounting for about 2–7% of the total number of cells in humans, endothelial cells can be found in any tissue that is vascularized. The average human has approximately 0.6–60 × 10^12^ endothelial cells, and given that the average surface area of a single endothelial cell is 20 × 60 µm, if endothelial cells were to be placed one next to the other, they could cover at minimum 720 m^2^, which is about the size of 4 average houses in the U.S. [1, 46–49]! Endothelial cells, of course, are not located in a single location in the body, but are present in every tissue that is vascularized, particularly in the lung where the largest capillary network is found [50]. As such, due to their wide distribution and essential physiological functions, endothelial cells can significantly contribute to numerous diseases. In cancer, for example, endothelial cells promote vessel formation and growth for oxygen and nutrient transport to support the proliferation of cancer cells, resulting in tumor growth and ultimately damage to the surrounding tissue [37–39]. In patients with diabetes, hyperglycemia causes a decrease in nitric oxide production and activity, which causes endothelial dysfunction that leads to atherosclerosis [40–42]. Chronic inflammation is another condition that damages endothelial cells and results in cardiovascular dysfunction [43–45]. In essence, endothelial cells will associate and contribute to a wide range of diseases since they are part of the cardiovascular system and essential for numerous physiological functions in the body (Fig. 1B). As such, therapies targeted to endothelial cells could prevent or treat numerous diseases.

## Nucleic Acid Therapeutics for Endothelial Cell Dysfunction

Delivery of nucleic acids, which have the capacity to modulate gene expression, to endothelial cells could be strategic in treating a broad range of diseases. The goal of gene therapy is to modulate gene expression of specific cells in the body to prevent, mitigate, or treat disease. Current technologies can alter gene expression at the DNA or mRNA level. Genome editing can render long-term gene correction that persists with the lifespan of the edited cell [62, 63]. Regardless of the strategy employed, gene therapy requires intracellular delivery of anionic and macromolecular nucleic acids, which do not readily traverse the hydrophobic cell membrane lipid bilayer. While various nucleic acid delivery technologies have been developed, this review focuses on the application of lipid nanoparticles (LNPs) for delivery to endothelial cells.

FDA approval of the LNP nucleic acid therapeutic patisiran (2018) and COVID-19 vaccines BNT162b2 and mRNA-1273 (2021, 2022) have paved the way in understanding baseline LNP pharmacokinetics and pharmacodynamics in humans [64]. Many studies have found LNPs to be effective, generally safe, and well-tolerated [65–67], making LNPs an attractive delivery platform. In general, LNPs comprise four key elements: an ionizable lipid, helper lipid, cholesterol, and poly(ethylene glycol) (PEG)-functionalized lipids. Ionizable lipids typically exhibit a *p*K_a_ < 7 and are therefore deprotonated (neutral) during circulation, which enhances the safety profile of lipids compared to permanently cationic lipids [68]. Accordingly, LNPs are formulated in acidic conditions (pH < 5) such that the ionizable lipid is protonated (cationic) and able to complex and condense anionic nucleic acids. Inclusion of helper lipids, cholesterol, and PEG-lipids at optimized ratios promotes LNP delivery efficiency, stability, and circulation time, as discussed in other reviews [69–71].

Given the broad capacity of LNPs to package, transport, and deliver nucleic acids, this section examines the types of nucleic acid developed for treating diseases involving the endothelium, with a focus on reports where an *in vivo*, disease-ameliorative effect was observed. An overview of therapeutic nucleic acids is provided in Table I.

**Table I:** Summary of Nucleic Acid Cargo Classes

Class	Description	Example Applications
DNA	• broad capacity to overexpress and/or silence genes of interest due to versatility • requires nuclear delivery • viral delivery enables long-term expression; non-viral delivery enables transient delivery but is not efficient in quiescent cells	• delivery of genes that rescue/mitigate endothelial dysfunction • knockout/correct pathogenic genes by encoding for CRISPR/Cas9 • modulate transcription by encoding for deactivated Cas9
mRNA	• similar to DNA, also exhibits versatile capacity to overexpress and/or silence genes of interest • does not require delivery into the cell nucleus • transient protein expression	• similar application as DNA, but gene expression is more transient • transient and local growth factor expression
saRNA	• encodes protein of interest and replicase, which replicates the saRNA	• applications have focused on vaccines due to immunogenicity
circRNA	• synthetic circRNAs exhibit greater stability against exonucleases compared to linear mRNA	• endogenous circRNA act as miRNA sponges and modulate gene dynamics • can also interact with proteins and modulate cell viability
siRNA	• triggers RNA interference (RNAi) • highly specific for cognate RNA strand	• silencing of endothelial cell pathways to reduce tumor burden • silencing of inflammation-associated pathways to attenuate fibrosis/tissue injury • attenuation of atherosclerosis
shRNA	• triggers RNAi • can be encoded in a DNA vector; thus, can be transient or long-term (viral delivery) and multiplexed	• tissue-specific promoters for tissue-specific RNAi • similar applications as siRNA
miRNA	• triggers RNAi • requires only partial complementarity to mRNA; thus can modulate multiple mRNAs	• delivery of hypoxia-upregulated miRNA to induce angiogenesis • delivery of anti-inflammatory miRNA to attenuate atherosclerosis
ASO	• can trigger RNAi or alternative splicing of mRNA • can achieve similar *in vivo* RNAi as siRNA • because ASOs are single-stranded, may be simpler and less costly than siRNA (double-stranded)	• similar applications as siRNA • alternative splicing; e.g., re-inclusion of normally excluded exons to rescue deficient protein expression

### DNA

DNA vectors perhaps wield the broadest flexibility in cargo and gene modulation, with the capacity to overexpress and/or silence genes of interest (e.g., encoding for short hairpin RNA or CRISPR/Cas9). Moreover, specific promoters can be included to restrict gene expression to certain tissues. In the case of LNP-mediated delivery, the DNA cargo typically takes the form of a plasmid. However, a major obstacle to non-viral, LNP-mediated DNA delivery is that nuclear localization of plasmid is required for protein expression (72), which does not efficiently occur in post-mitotic cells. While peptide nuclear localization sequences can augment plasmid gene delivery in quiescent cells [73, 74], endothelial cells can proliferate during disease and inflammation [75, 76], providing an avenue for non-viral gene delivery to these cells. Here, examples of DNA gene delivery to the *in vivo* endothelium are reviewed.

Intravenous delivery of plasmids encoding human indoleamine-2,3-dioxygenase (hIDO), gated under an endothelial-specific endothelin-1 promoter, resulted in hIDO expression in pulmonary endothelial cells. In a model of lung transplant ischemia–reperfusion injury, hIDO treatment reduced lung permeability and inflammation and protected function of the transplanted lungs [77]. *Ex vivo* transduction of the corneal vasculature with Bcl-xL improved graft survival in a corneal transplantation model [78].

In a model of hypertension, HO-1 gene delivery under control of the endothelial-specific VE-cadherin promoter mitigated increases in blood pressure and blood markers of inflammation [79].

Delivery of plasmid encoding VEGF to vascular endothelial cells in a model of carotid artery injury enhanced recovery and inhibited neointimal hyperplasia [80]. Similarly, VEGF plasmid delivery to the endothelium in a rabbit model of balloon angioplasty-induced injury attenuated intimal thickening [81]. Delivery of eNOS plasmid directly to iliac artery endothelial cells via a stent promoted re-endothelialization and mitigated neointimal hyperplasia in a rabbit model of restenosis [82]. In another application, viral delivery of eNOS to the thoracic aorta endothelium and small renal arteries mitigated hypertension and renal scarring in a model of renal failure [83].

In addition to overexpression of therapeutic genes, DNA vectors have also been used to knockout pathogenic genes by encoding for CRISPR/Cas9. A missense mutation of collagen 8A2 leads to Fuch’s endothelial corneal dystrophy (FECD). In a genetic mouse model of early-onset FECD, CRISPR/Cas9-mediated disruption of the *Col8a2* start codon in the corneal endothelium mitigated disease burden [84]. Deactivated Cas9 (dCas9) can be used to “ferry” gene activators or repressors proximal to the gene of interest, modulating gene expression without altering the host genome [85]. Delivery of a *Sox2*-activating dCas9 construct to the corneal endothelium improved wound healing and endothelial regeneration in a model of corneal endothelial injury [86].

DNA encoding of CRISPR/Cas9 to install base edits has been described in other applications. Chen *et al.* intratumorally delivered NPs carrying plasmids encoding for a base editor and sgRNA into tumors *in vivo*, which expressed eGFP only when a stop codon is correctly edited [87]. Moreover, hydrodynamic injection of plasmids encoding for Cas9 and sgRNAs, as well as template DNA, was able to facilitate GFP hepatocyte knockin *in vivo* [88].

### Messenger RNA (mRNA)

Similar to DNA, mRNA can also achieve different modes of gene modulation. Some examples include: therapeutic protein overexpression by encoding the protein of interest, or gene knockout by encoding for CRISPR/Cas9. In non-viral gene delivery, a major advantage of mRNA compared to DNA cargoes is that mRNA does not require delivery into the cell nucleus. As a result, mRNA can achieve robust gene delivery even in challenging cell types [89–91]. Moreover, advances in synthetic bases, mRNA manufacturing capacity, and codon optimization have made clinical-scale mRNA production feasible [92].

While new formulations of LNPs have enabled mRNA delivery to liver sinusoidal [93], splenic [94], and lung [91, 95, 96] endothelial cells, these reports have primarily utilized reporter mRNAs (e.g., luciferase, Cre recombinase) and not therapeutic mRNAs in disease models. This may be due to several reasons, including: the field of LNP-mediated mRNA delivery to endothelial cells is still being developed, the need for sustained transgene expression in chronic diseases that affect the endothelium necessitates alternative vectors, and the current challenge of delivering mRNA to non-hepatic tissues. Another recognized challenge is the limited tropism of current LNPs that may limit the capacity to deliver mRNA to endothelial cells within multiple or specific organs (e.g., heart, aorta), which may be required to combat systemic diseases. While mRNA results in transient protein expression (~ 1 wk), there are still applications that may benefit from an acute intervention that engenders long-term, therapeutic effects [97]. Due to the lack of reports of direct therapeutic mRNA delivery to endothelial cells, this section will focus on applications in which endothelial cell behavior is modulated by mRNA delivery to treat disease *in vivo*.

Szőke *et al*. developed an mRNA-LNP for the treatment of lymphedema, which may arise due to injury to the lymphatic vessels. A single intradermal injection of VEGF-C mRNA-LNPs induced proliferation of lymphatic endothelial cells and reduced limb swelling in a genetic model of lymphedema [98]. While the initial transfection was not specific to lymphatic endothelial cells, local transfection likely led to VEGF-C secretion that acted on proximal lymphatics. Intradermal delivery of VEGF-A mRNA enhanced oxygenation and accelerated wound healing in a mouse model of diabetic wounds [99].

Aging can lead to blindness due to abnormal growth of new blood vessels. An important source of VEGF in the eye are retinal pigment epithelial cells. Ling *et al.* subretinally injected CRISPR/Cas9 mRNA, packaged inside lentivirus, against *Vegfa*, which led to knockout of *Vegfa* in the retinal pigment epithelium and reduced the burden of laser-induced choroidal neovascularization [100].

Direct injection of mRNA into murine hearts led to robust transfection of endothelial cells, cardiomyocytes, and smooth muscle cells. Interestingly, mRNA encoding VEGF-A improved survival in a mouse model of myocardial infarction more efficiently than DNA. This was attributed to sustained, DNA-mediated VEGF-A expression leading to greater vascular leakage and highlights the advantage of acute transgene expression afforded by mRNA in certain applications [101].

In addition to gene knockout, mRNA delivery of base editors could be deployed to install single-nucleotide edits. Base editor delivery to endothelial cells have yet to be described, but have been applied to other organ systems. Villiger *et al*. delivered the *Sa*KKH-CBE3 base editor in mRNA form using LNPs, which installs a therapeutic C-to-T genetic change to correct a pathogenic mutation in *Pah*^enu2^ mice hepatocytes [102]. LNPs can also facilitate homology-directed repair for genetic editing of longer DNA sequences. Farbiak *et al*. delivered Cas9 mRNA, sgRNA, and template DNA intratumorally and observed *in vivo* editing of tumor fluorophore expression (103).

### Self-Amplifying RNA (saRNA)

As their name suggests, saRNA are able to propagate through encoding of a viral replicase. Within the saRNA are sequences that encode for replicase components, a subgenomic promoter, and a gene of interest downstream of the promoter. When saRNA (positive strand) is delivered, replicase is translated that makes complementary saRNA (negative strand), which then acts as a template for replicase to synthesize either more positive strand saRNA or gene of interest RNA [104]. Amplification of both positive strand saRNA and gene of interest RNA therefore enables greater protein expression at significantly reduced doses compared to normal mRNA [105].

The inherent immunogenicity of saRNA has led to their predominant application in vaccines. saRNA is considered a self-adjuvant due to activation of interferon responses [106, 107]. Indeed, lower doses of saRNA are able to elicit greater antibody titers compared to higher doses of DNA [108]. There is currently considerable interest in developing saRNA for COVID, rabies, and cancer vaccines, with many active clinical trials [109, 110]. Non-vaccine applications remain to be developed, although alternative vectors with reduced immunogenicity may be preferred over saRNAs.

### Circular RNAs (circRNAs)

Endogenous circRNAs play various roles in regulating gene expression, which may motivate new therapeutic applications. circRNAs can act as miRNA “sponges,” and by acting as decoy binding sites to miRNA, they can enable greater expression of miRNA targets that are otherwise degraded. For example, ciRS-7 (cirRNA sponge for miRNA-7) is highly expressed in human and mouse brains and contains multiple sites for miRNA-7 binding, which does not cause ciRS-7 degradation. Cells expressing ciRS-7 exhibited reduced knockdown of known targets of miRNA-7, *SNCA*, *EGFR*, and *IRS2*, compared to empty vector cells [111]. Another circRNA that also binds to miRNA-7, CDR1as, is highly expressed in the brain along with miRNA-7. In cultured cells, knockdown of CDR1as resulted in increased knockdown of miRNA-7 targets [112].

circRNA can also regulate gene expression through interactions with proteins. As an example, circANRIL (circular antisense non-coding RNA in the INK4 locus) binds to PES1, a protein which impairs ribosome formation and triggers apoptosis and reduces proliferation. This particular pathway may be important in protection against atherosclerosis [113].

Recently, synthetic circRNAs have been developed to enhance stability against exonucleases and prolong expression of delivered genes. *In vitro*, circRNA exhibited greater and longer luciferase activity compared to modified and unmodified linear mRNA [114], providing evidence for the enhanced stability of circRNA. Moreover, unmodified circRNA exhibits reduced immunogenicity compared to unmodified linear mRNA; *in vivo*, local injection of LNPs delivering hEpo-encoding circRNA resulted in a greater proportion of serum hEpo (~ 50%) at 42 h relative to levels at 6 h compared to linear, modified mRNA (~ 20%) [115]. A recent report designing and optimizing circRNAs observed durable hEpo expression in mice up to 96 h using circRNA, whereas hEpo expression decreased after 24 h using mRNA [116]. Engineering and delivery of circRNA is a developing research area; ostensibly, strategies described to deliver DNA and mRNA to endothelial cells could be deployed using circRNAs in which stable expression is required.

### Small Interfering RNA (siRNA)

siRNA has enabled tailorable and precise gene silencing of target mRNA. Synthetic siRNA is typically delivered as a duplex comprising a sense and antisense strand. Once in the cytoplasm, siRNA complexes with RNA interference (RNAi) enzymes, during which the passenger strand is degraded and the mature RNA-induced silencing complex (RISC) forms. This RISC is now capable of degrading mRNA that is recognized by the guide strand [117–119]. Gene knockdown can be long-term, persisting for approximately a month in humans [64, 120]. Endothelial gene targets for siRNA therapy in various disease contexts are reviewed here.

Delivery of siRNA against VEGFR-1, DLL4, or CD31 to pulmonary endothelium reduced tumor and metastases burden in a model of lung cancer [121–123]. Silencing of angiopoietin-2 in lung endothelium improved lung function and survival, and had distal renoprotective effects in mouse models of sepsis [124]. Broad delivery of CD31, but not Tie2, siRNA to endothelial cells reduced tumor burden in an orthotopic prostate cancer model [125]. Systemic delivery of VEGFR-2 and PLXDC1 siRNA to tumor endothelium reduced tumor burden [126, 127], and STAT3 siRNA directed to bone marrow endothelium in a model of bone metastasis prolonged survival in tumor-bearing mice [128].

In an acute, LPS-induced mouse model of inflammation, broad delivery to endothelial cells of siRNA against NF-κB p65 (RelA) reduced inflammation in kidney tissue [129]. During ventilator-induced lung inflammation, delivery of calpain-1 siRNA to pulmonary endothelium reduced polymorphonuclear neutrophil infiltration into bronchoalveolar lavage fluid, an indicator of inflammation [130]. siRNA knockdown of ICAM-1 in cardiac microvascular endothelial cells attenuated cardiac infarct size and fibrosis, and improved cardiac function after myocardial ischemia–reperfusion injury [131]. Similarly, simultaneous delivery of siRNA against ICAM-1, ICAM-2, VCAM-1, E-selection, and P-selectin to endothelial cells attenuated inflammation, immune cell infiltration, and aortic plaque development in an accelerated inflammation model [132].

Delivery of VEGFR-2 siRNA to endothelial cells in a model of portal hypertension reduced the severity of pathological angiogenesis, portosystemic collateralization, and collateral blood flow [133]. In a similar application, COX-1 siRNA, delivered to liver sinusoidal endothelial cells, also reduced portal pressure in CCl_4_-induced cirrhotic mice [134].

During a mouse model of high-cholesterol diet-induced atherosclerosis, delivery of siRNA against LOX-1 to the aortic endothelium attenuated plaque development and macrophage infiltration [135].

In a dexamethasone-induced murine model of ocular hypertension, an intracameral injection of siRNA against tricellulin led to reduced tricellulin and ZO-1 expression in Schlemm’s canal endothelial cells and reduced intraocular pressure [136].

### Short Hairpin RNA (shRNA)

shRNA, also used for RNAi therapy, comprises a duplexed passenger and guide strand “stem” region connected via a short linker “loop” and causes degradation of target mRNA. Similar to siRNA, shRNA complexes with RNAi enzymes during which the passenger strand and loop region are removed, forming the RISC [117]. An advantage of shRNA over siRNA is its ability to be encoded in a DNA vector, enabling “all-in-one” simultaneous knockdown(s) and transgene expression in a single vector [137, 138]. DNA encoding also enables viral transduction of shRNA for durable knockdown and engineered promoters for tissue-specific shRNA expression (138–141). Here, reports delivering shRNA to the endothelium with an observed *in vivo* effect are described.

In a mouse model of atherosclerosis, delivery of DNA encoding RAGE-shRNA to activated endothelial cells reduced the burden of atherosclerotic plaques and circulating inflammatory cytokines [142]. Similarly, viral delivery of PTP1B or METTL3 shRNA to aortic endothelium mitigated the development of atherosclerotic lesions in models of atherosclerosis [143, 144]. Knockdown with PDGF-A shRNA in the aortic endothelium of diabetic mice overexpressing BMP4 (an inflammatory cytokine upregulated in atherosclerotic plaques) improved endothelial-dependent relaxation [145]. Viral transduction of TPRM2 shRNA in aortic endothelial cells improved aortic vasorelaxation in obese mice [146].

In an interesting study, Stimac *et al.* compared the tumor-killing efficiency of endoglin shRNA with either a constitutive or endothelial cell-specific promoter. While both variants dampened tumor growth and induced significant tumor necrosis *in vivo*, there were no statistically significant differences in performance between the two variants [141].

Viral transduction of ATG7 shRNA, with expression constrained to retinal vasculature, protected against endothelial dysfunction in a model of diabetic retinopathy [140].

Knockdown with salusin-*β* shRNA in the coronary, pulmonary, and mesenteric arteries of a rat model of chronic heart failure improved cardiac function and vascular remodeling [147].

### MicroRNA (miRNA)

miRNA presents another strategy for RNAi, and is processed within the cell nucleus from a larger, stem-loop structure (primary miRNA) into a smaller structure (pre-miRNA) that is exported into the cytoplasm [148, 149]. There, the pre-miRNA is further processed into double-stranded miRNA, and during loading onto the miRNA-induced RISC (miRISC), the passenger strand is discarded [148, 149]. The miRISC is now capable of repressing translation of mRNA recognized by the guide strand. A key feature of miRNA over siRNA is that miRNA needs only partial complementarity to mRNA to facilitate RNAi. Indeed, a single miRNA sequence can modulate multiple mRNAs [150]. Here, some applications of therapeutic miRNA delivery to the endothelium are reviewed.

Delivery of miRNA-210, a miRNA that is physiologically upregulated during hypoxia and induces angiogenesis, to cerebral vascular endothelial cells led to increased VEGF mRNA levels, angiogenesis, and animal survival in a middle cerebral artery occlusion mouse model [151, 152]. In another therapeutic application of ischemia injury, delivery of miRNA-126-3p to endothelial cells in ischemic muscle augmented blood flow and vessel density in a model of chronic ischemia [153]. Mechanistically, miRNA-126-3p represses negative regulators of VEGF signaling.

miRNA-146a and miRNA-181b were hypothesized to be protective in a model of high-fat diet-induced atherosclerosis, due to their anti-inflammatory effects. While miRNA delivery was shown in *ex vivo*, and not in *in vivo* aortas, the report utilized a thioaptamer that had been described to recognize E-selectin that is expressed on inflamed endothelium after intravenous administration [128]. *In vivo* treatment with either miRNA-146a or -181b reduced plaque formation and macrophage infiltration [154].

In a wound-healing application, outgrowth endothelial cells (OECs) were first loaded with miRNA-155-5p and miRNA-302a-3p, which promote endothelial survival during hypoxia and cell proliferation, and then transplanted into wounds. The miRNAs were immobilized onto gold nanorods, and release of each miRNA was triggered by laser irradiation at different settings. Interestingly, release of miRNA-302a-3p first, followed by release of miRNA-155-5p, accelerated healing compared to unloaded cells and the reverse miRNA release order [155].

During asthma, miRNA-1 dampens eosinophil recruitment by suppressing the expression of inflammation- and adhesion-related receptors. Here, miRNA-1 delivery was achieved using a lentivirus, and a VE-cadherin promoter was used for endothelial-specific miRNA-1 expression. In a model of asthma, intranasal delivery of this lentivirus reduced eosinophil infiltration, airway inflammation, and airway resistance [156].

miR-20a modulates multiple pathways including angiogenesis and DNA synthesis and replication, and is significantly downregulated in liver sinusoidal endothelial cells (LSECs) during liver cancer. Targeted delivery of miR-20a to LSECs significantly reduced liver tumor burden in a model of colorectal cancer liver metastasis [157]. During certain forms of liver injury, miRNA-30c, which modulates LSEC proliferation, migration, and angiogenesis, is downregulated. Delivery of miRNA-30c to LSECs significantly attenuated liver fibrosis [158].

### Antisense Oligonucleotides (ASOs)

ASOs are synthetic, single-stranded oligomers capable of gene suppression or mRNA transcript modulation [159]. To suppress gene expression, “gapmer” ASOs are generally designed to contain a DNA segment flanked by RNA-based segments that are complementary to the mRNA target of interest. RNASEH1, which recognizes RNA–DNA heteroduplexes, catalyzes the degradation of the cognate mRNA after ASO hybridization [159, 160]. To facilitate transcript modulation, ASOs are engineered to recognize specific exons but not trigger the RNASEH1 response. Rather, the hybridized ASO acts as a steric “block” that causes alternative splicing of mRNA to exclude or include exons [159, 161]. Clinically approved ASOs include inotersen and nusinersen; notably, these ASOs are injected without the use of nanoparticle delivery systems.

In terms of gene inhibition, both siRNAs and ASOs can have similar *in vivo* efficiency when optimized [162]. Clinical production of ASOs, which are single-stranded, may be simpler and less costly compared to double-stranded siRNAs. The capacity to incorporate synthetic bases may also augment the stability and half-life of ASOs [159, 163]. Here, ASOs with a therapeutic effect via modulating genes in endothelial cells are reviewed.

ICAM-1 is upregulated during inflammation on endothelial cells and utilized by immune cells to interact with and extravasate from the endothelium [164]. Intravenous infusion of ASOs against ICAM-1 reduced ICAM-1 expression in kidney endothelium and protected renal function subjected to ischemia injury alone or in combination with transplantation [165, 166]. Similarly, administration of an ICAM-1 ASO alone and in combination with an anti-LFA monoclonal antibody prolonged allograft survival in a mouse model of heart transplantation [167]. *Ex vivo* knockdown of endothelin-1, which is expressed in endothelial cells, reduced the burden of graft coronary artery disease in a rat model of allograft [168].

Direct injection into the anterior chamber of the eye of siRNA or ASOs against Cx43 resulted in reduced Cx43 expression in the corneal endothelium and accelerated healing after scrape injury [169]. Notably, both siRNA and ASO molecules performed equally well in this model.

In an interesting approach, direct delivery of PDGF ASO to the coronary endothelium using ASO-coated stents significantly inhibited restenosis in a porcine model [170].

While ASOs have been extensively developed to silence gene targets in endothelial cells, their use for alternative splicing in these cells has not been as widely described. As such, we will highlight the FDA-approved ASO nusinersen, which mediates therapeutic RNA splicing to treat spinal muscular atrophy; other splice-inducing ASOs are discussed in greater depth in these reviews [171, 172]. During normal conditions, *SMN1* is the principal source of SMN protein. While there is a second *SMN2* gene, it contains a C > T mutation in exon 7 that leads to exclusion of this exon and an unstable protein product [173]. Mutations in *SMN1* can disrupt survival motor neuron (SMN) protein expression, leading to spinal muscular atrophy. The therapeutic goal of nusinersen was to reinclude exon 7 in *SMN2* transcripts, thereby leading to functional SMN protein translation. This was achieved by designing the ASO to hybridize with *SMN2* at a site that blocks RNA-binding of hnRNP, which normally represses exon 7. Blocking this repressor therefore promotes inclusion of exon 7 and rescue of SMN protein expression via *SMN2* [174].

## Strategies to Direct Lipid Nanoparticles to Endothelial Cells

Various chemical and non-chemical methods have been developed to deliver LNPs into endothelial cells of various organ systems. This section will review these strategies and a summary is presented in Table II.

**Table II:** Strategies and Compositions to Mediate Nucleic Acid Delivery to Endothelial Cells

Strategy	Major Targeting Component	Organ/Tissue Targeted	Example Refs
formulating nanoparticles with cationic lipids or polymers	7C1 lipid-polymer	lung, heart, kidney, liver, spleen	[94, 121, 175]
	poly(β-amino esters) lipid-polymer	lung	[91, 176]
	poly(amido amine) or poly(propylenimine) lipid-dendrimers	lung, liver	[177, 178]
	AtuFECT01 lipid	lung, heart, liver	[122, 179]
	DMAPAP lipid	activated endothelium	[142, 180]
	DDAB lipid	lung	[130, 181]
	EPC lipid	lung	[181]
	DOTAP lipid	lung	[181, 182]
anchoring targeting ligands to nanoparticle surface	GALA peptide	lung	[123]
	mannose	liver	[93]
	PECAM-1 antibody	lung	[96]
	PV1 antibody	lung	[183]
	VCAM-1 antibody	activated endothelium	[129]
	P-selectin peptide	activated endothelium	[142, 180]
	chondroitin sulfate	liver	[157]
incorporation of helper lipids or cholesterol to nanoparticle formulations	anionic DSPG	liver	[184]
	oxidized cholesterol	liver	[185]
mechanically directing nanoparticles to vessel wall	mechanical stent	local delivery (iliac artery)	[82]
ultrasound-targeted microbubble destruction	DSTAP lipid	hindlimb muscle	[153]

### Cationic Lipids

Intravenous administration of lipid nanoparticles has been the most common route to deliver therapeutic genes to endothelial cells, as these cells are located in the inner surface of blood vessels and are in direct contact with the blood. However, when nanoparticles enter the blood circulation, they are often eliminated by cells of the reticuloendothelial system or preferentially transfect hepatocytes, which prevents nanoparticles from reaching and transfecting endothelial cells [69, 94, 186–189]. As such, strategies have been developed to bypass hepatocytes in order to reach and transfect endothelial cells. Among the strategies developed, the use of cationic lipids or polymers to generate nanoparticles that target endothelial cells has made considerable progress. Dorkin *et al.* showed that incorporation of the permanently cationic lipid, DOTAP, to LNPs that otherwise target the liver could be redirected to transfect pulmonary endothelial cells [182]. Dorkin *et al.* also found that liver-targeted lipid nanoparticles, which are nanoparticles that preferentially transfect hepatocytes on their own, specifically C12-200, cKK-E12, and 503O13 nanoparticles, could have a shift in their tropism and transfect pulmonary endothelial cells by incorporating the lipid DOTAP in their formulation [182]. This finding was later expanded by Cheng *et al.* to include additional cationic lipids, such as DDAB and EPC, to other liver-targeted nanoparticles like 5A2-SC8 and DLin-MC3-DMA to enable transfection of pulmonary endothelial cells [181]. The mechanism by which these nanoparticles target and deliver nucleic acids to the lung may be due to association with a protein or group of proteins that binds to the surface of the nanoparticles and selectively delivers them to the pulmonary endothelium [95, 190]. For example, lipid compositions can shift the protein corona abundance away from ApoE, which is a recognized driver of hepatocyte LNP uptake and transfection [191].

Knowing that lipid nanoparticles can be directed to the pulmonary endothelium by introducing cationic lipids in their formulation established one of the first rational design strategies to target endothelial cells. This finding could accelerate the production of targeted nanoparticles to the endothelium, as most nanoparticles generated prior to this discovery were fabricated with lipids obtained from tedious library screens or novel chemical synthesis. Coincidentally, most lipids generated through those approaches turned out to be cationic. AtuFECT01, for example, is a cationic lipid that was derived from a novel chemical synthesis that was used to formulate nanoparticles with siRNA to knockdown genes of endothelial cells [179]. The nanoparticles made with the AtuFECT01 lipid were capable of transfecting endothelial cells in various organs, including the lung, heart, and liver [179]. These nanoparticles were later optimized to become selective to pulmonary endothelial cells by increasing the amount of AtuFECT01 lipid and introducing cholesterol in their formulation [122].

Another cationic lipid that was derived from a library screen and used in the formulation of siRNA containing nanoparticles to target endothelial cells is 7C1 [121, 175]. This lipid, which was obtained from the conjugation of alkyl chains to low molecular weight polyethylenimine (PEI), formed nanoparticles that were highly selective for endothelial cells of mice and non-human primates, and was capable of transfecting the endothelium of various organs, including the lung where they had the highest transfection efficiency [121, 175]. These 7C1 nanoparticles were later optimized to deliver Cas9 mRNA and sgRNA to splenic endothelial cells [94]. Khan *et al*. and Kaczmarek *et al*. similarly derived cationic lipids from library screens that generated nanoparticles capable of transfecting endothelial cells *in vivo* [91, 176, 178]. Khan *et al*. derived a lipid from the conjugation of alkyl chain to branched PEI that delivered siRNA to pulmonary endothelial cells, while Kaczmarek *et al*. derived a poly(β-amino ester)-based lipid that delivered mRNA to the lung endothelium [91, 176, 178]. Constantinescu *et al*. and Cao *et al*. also utilized DMAPAP and DSTAP cationic lipids for nucleic acid delivery to endothelial cells, and more recently, Qiu *et al.* synthesized a library of lipids to demonstrate that lipids containing an amide bond linker formed nanoparticles that selectively delivered mRNA to the lung, as opposed to lipids containing an ester bond linker which formed nanoparticles that targeted the liver [95, 153, 180]. Lastly, lipofectin, which is a commercially available reagent containing cationic lipids, has been used to enhance delivery of ASOs to vascular cells *in vivo* [165, 166].

### Targeting Ligands

Lipid nanoparticles have also been modified with targeting motifs on their surface or through the addition of cholesterol or non-cationic helper lipids in order to enable and improve selectivity for endothelial cells. Since endothelial cells express unique surface molecules such as sugars and proteins, lipid nanoparticles have been coated with molecules that target endothelial surface molecules. For example, Kusumoto *et al*. developed a lipid nanoparticle coated with GALA peptides on their surface to selectively target pulmonary endothelial cells [123]. The GALA peptides were capable of directing the nanoparticles to endothelial cells by targeting the sialic acid-terminated sugar chains on the pulmonary endothelium, which subsequently delivered the encapsulated nucleic acids to the endothelial cytosol via endosomal membrane fusion [123].

Similarly, Kim *et al*. reported that the addition of mannose to lipid nanoparticles enabled selective RNA delivery to liver sinusoidal endothelial cells, as mannose receptors are preferentially expressed in the liver endothelium and therefore nanoparticles containing mannose on their surface would be scavenged by liver endothelial cells [93]. In the same form, Parhiz *et al*. and Marquez *et al*. demonstrated that the addition of PECAM-1 antibody or chondroitin sulfate allowed the selective delivery of LNPs to endothelial cells by targeting proteins ubiquitously expressed on the endothelium [96, 157].

Other reports have demonstrated that the addition of antibodies or binding peptides that target adhesion proteins on an activated endothelium directs LNPs to endothelial cells from different organs. One example of such work was produced by Kowalski *et al*. who showed that addition of anti-VCAM-1 antibodies to the surface of SAINT-O-Some lipid nanoparticles facilitated the delivery of siRNA to inflamed renal endothelial cells [129]. In the brain, nanocarriers functionalized with antibodies that recognize VCAM-1 successfully delivered thrombomodulin (TM)-encoding mRNA and mitigated TNFα-induced cerebral edema in a rat model [192]. Uptake of anti-VCAM-1 immunoliposomes was further evaluated in cultured ECs and reported to occur via clathrin-mediated endocytosis [193]. Li *et al.* leveraged the fact that endothelial cell membranes in lung capillaries are enriched for caveolae. Modification of LNPs with an antibody that recognizes PV1, a caveolae-associated protein, significantly augmented lung mRNA delivery and transgene expression (183).

For adenovirus, one strategy is to utilize bispecific antibodies that recognize a virus domain for antibody attachment (e.g., knob, capsid) and angiotensin-converting enzyme (ACE), which has been extensively for adenovirus vectors to deliver nucleic acids to pulmonary endothelial cells [194–198]. Using this scheme, Morecroft *et al.* observed 50-fold higher pulmonary endothelial luciferase expression and an 87% reduction in liver expression of ACE-conjugated adenovirus compared to non-targeted vectors [194]. Similarly, adenovirus delivery of ACE-targeted endothelial nitric oxide synthase (AdeNOS) to the carotid artery of stroke-prone hypersensitive rats significantly reduced blood pressure compared to untargeted virus, underscoring the biological efficacy of retargeting of viruses to ACE [195].

### Protein Considerations: Intra- *versus* Extracellular Delivery

When using targeting ligands to direct nanoparticles to the endothelium, it is important to recognize that endothelial cells have different internalization efficiencies depending on the type of molecule targeted and composition of the targeting ligand. For example, endothelial cells efficiently internalize antibodies that recognize ACE [199] but poorly internalize single PECAM-1 antibodies [200, 201]. The uptake of PECAM-1 antibodies, however, can be enhanced by conjugation of biotin with streptavidin and are transported intracellularly through an epitope-specific pathway [201–203]. Similarly, conjugates utilizing antibodies that bind to ICAM-1 are endocytosed at higher levels than conjugates targeted to PECAM-1, and their internalization efficiency can be tuned by adjusting conjugate size and shape. However, the conjugates must be multimeric because monomeric versions are not internalized by endothelial cells [204–206]. In general, while free antibodies targeted to adhesion molecules of endothelial cells are not readily internalized by the endothelium, functionalization with multiple antibodies (i.e., multivalency) improves endothelial targeting to the lung, particularly to nanoparticles with a diameter of 100 nm and above [207].

The poor internalization of monovalent PECAM-1 antibodies has been capitalized for other applications to anchor extracellular protein therapeutics within the endothelial lumen. TM fused with a monovalent single chain variable fragment (scFv) of PECAM-1 antibody and urokinase plasminogen activator (scFv/uPA) augments thrombin activity and biodistribution to pulmonary vasculature compared to soluble TM in a mouse model of acute lung injury [208]. This targeting capability was further improved in another study where endothelial targeting of scFv/TM to ICAM-1 facilitated ∼15-fold greater activated protein C (APC) levels than its PECAM-1-targeted counterpart. This increased activity may be due to the proximity of ICAM-1 to EPCR, which is exposed in the apical membrane and a key cofactor of TM/APC [209].

### Biomechanical Factors

Biomechanical factors, such as blood flow, along with carrier physical properties and mode of internalization, can also influence targeting and uptake efficiencies by endothelial cells. It has been demonstrated that blood flow encourages the uptake of spherical antibody nanocarriers by endothelial cells in the absence of stress fiber formation, but actin stress fiber development and endothelial alignment with flow reduces uptake of nanocarriers functionalized with antibodies that recognize PECAM-1 and ICAM-1 [200, 210, 211]. This phenomena should be considered when directing nanoparticles to selective blood vessels in the body, as endothelial cells in the arterial vasculature elongate during adaptation to high rates of unidirectional flow and capillary endothelial cells exposed to low or oscillating flow obtain morphology that is similar to cultured endothelial cells. This means that targeted nanocarriers will have lower levels of nanoparticle internalization in arterial relative to capillary vessels due to the cell structure [200].

Carrier geometry also influences endothelial targeting and the rate of endocytosis and lysosomal transport within endothelial cells. For example, elliptical disk-shaped carriers have higher specificity *in vivo* than those with a spherical shape, but have lower endocytic efficiencies [212]. Avoidance of the reticuloendothelial (RES) system can be achieved by rod-shaped particles containing anti-ICAM-1 antibodies by taking advantage of the cellular hitchhiking effect [213]. Similarly, circulation time is affected by the geometry of the carrier and its alignment with flow, as it has been demonstrated that filomicelles that are long and flexible have longer circulation time periods than those that are small and rigid [214]. The size of the carrier and mode of internalization also affects targeting and intracellular trafficking, as micron-size carriers have longer residency in pre-lysosomal compartments, while sub-micron carriers are transported to lysosomes more readily [212]. Nanoparticles coated with anti-ICAM-1 antibodies have been found to enter cells via endocytosis that bypasses the clathrin-dependent pathway to reach lysosomes, while PLVAP-targeted nanoparticles can be internalized by endothelial cells to reach caveola-derived endosomes through dissociation from caveolin-containing vesicles [215–217].

### Endothelial State and Carrier Interactions

The location of the target protein (e.g., ICAM-1) within the cell membrane, coupled with the mechanical properties of the carrier and disease alterations, can contribute to distinct delivery efficiencies when targeting endothelial cells. For example, while rigid nanocarriers with a diameter of 100 nm or greater can readily target adhesion molecules on endothelial cells such as ICAM-1, these same nanoparticles cannot target surface proteins in the endothelial caveolae, since the cutoff size to enter this area is about 50 nm [207, 218, 219]. Flexible nanoparticles have been demonstrated to reach endothelial caveolae even if their diameter is greater than 50 nm due to their mechanical deformability [218]. Overexpression of adhesion molecules during diseases, particularly during inflammation, can improve the delivery of nanocarriers to endothelial cells. ICAM-1 expression, for example, has been shown to be enhanced during systemic inflammation and improve the delivery of ferritin and polystyrene nanocarriers to pulmonary endothelial cells in mice [211, 220, 221].

APN and TEM-1 are also endothelial proteins that are upregulated in tumor microenvironments and could be targeted for the delivery of nanoparticles to endothelial cells for the treatment of cancer [221]. However, it is worth noting that the targeting ligand used for directing nanoparticles to the endothelium should be carefully selected to ensure it will not interfere with important biological functions of endothelial cells and induce adverse side effects. For example, the monoclonal antibody 273-34A enables liposomal delivery to the lung endothelium when conjugated to the surface of the particles by targeting thrombomodulin, which is a protein primarily expressed on the luminal surface of endothelial cells [221–225]. Similar antibodies have shown effective intracellular or surface delivery of nanoparticles to endothelial cells by targeting thrombomodulin [226]. However, thrombomodulin is a receptor of thrombin and, in combination with the plasma protein C, this protein converts thrombin into an anticoagulant enzyme. Therefore, antibodies targeted to thrombomodulin may interfere with the coagulation cascade and pose a risk of inducing thrombosis, making those antibodies unattractive for nanoparticle targeting [217, 221].

### Mechanical and Non-Cationic Lipid Methods

Beyond the use of targeting ligands, additional techniques have been implemented to deliver lipid nanoparticles to endothelial cells, such as the incorporation of cholesterol or non-cationic helper lipids into the formulation of LNPs. Paunovaska *et al*. recently reported that replacement of unmodified cholesterol with oxidized cholesterol from the formulation of cKK-E12 nanoparticles produced a five-fold improvement in the delivery of mRNA to liver endothelial cells [185]. Similarly, Pattipeiluhu *et al*. reported that replacement of neutral DSPC with anionic DSPG in the formulation of patisiran nanoparticles significantly enhanced transfection of liver endothelial cells [184]. Other strategies that have been reported to augment or facilitate transfection of endothelial cells are the use of larger diameter nanoparticles to prevent nanoparticle elimination from the circulation or the use of mechanical stents to locally deliver nanoparticles to the surface of blood vessels. Kim *et al*. and Khan *et al*. reported that nanoparticles with larger diameter sizes preferentially transfected liver endothelial cells over hepatocytes, likely because the nanoparticles were not filtered through the fenestrations of the liver vasculature, while Brito *et al*. reported that lipid nanoparticles immobilized on a stainless-steel stent achieved local transfection of endothelial cells [82, 93, 177].

### Navigating Intracellular Delivery

There are many extra- and intracellular barriers against nucleic acid delivery to endothelial cells; an overview is provided here and greater discussion is provided in these reviews [227, 228]. As discussed above, functionalization of LNPs with antibodies or specific lipids can mediate functional mRNA delivery to the lung endothelium [96]. Size, shape, and ligand avidity also contribute: 200–250 nm, but not 600–700 nm, PECAM-1 antibody-functionalized materials successfully delivered functional enzymes into lung endothelium; ICAM-1 antibody functionalization enhanced greater selectivity for diseased lung endothelium; and uptake of antibody-functionalized spheres was more efficient than polymorphous shapes [204, 205, 229].

After endocytosis, LNPs are then sequestered and trafficked into early endosomes, which acidify and mature into endolysosomes where LNPs are either exocytosed or degraded. Endosomal escape before lysosomal maturation is thus essential for successful nucleic acid delivery and is a major obstacle against delivery: < 2% of administered LNPs containing siRNA achieves endosomal escape [230]. While the “proton sponge” effect is thought to mediate endosomal lysis (due to buffering and water flux) for cationic polymers such as PEI [231], Gilleron *et al.* posit that their findings do not support this hypothesis for LNP delivery of siRNA [230]. Another suggested mechanism is that endosomal acidification drives protonation of the ionizable lipid. Now cationic, these lipids can interact with the lipids of the endosomal bilayer, disrupting and destabilizing the bilayer and enabling nucleic acid release [230, 232, 233]. Cholesterol may also contribute: replacement of cholesterol with β-sitosterol augments endosomal escape [234].

While most of the nucleic acids discussed earlier are active after cytoplasmic delivery, DNA further requires nuclear transport to be active. In addition to the peptide strategies discussed earlier, inclusion of binding site sequences can facilitate recruitment of transcription factors, which contain nuclear localization signals for nuclear entry [72].

## Clinical & Translational Perspectives

While LNPs encapsulating nucleic acids have successfully been translated into the clinic, many challenges remain in their implementation. Here we describe some of these challenges, highlighting some research efforts and drawing lessons from LNPs that are approved or have undergone clinical trials to answer them.

### Infusion-Related Reactions

LNP components can be recognized by the immune system and activate the complement cascade [235, 236], resulting in various infusion reactions. Complement activation-related pseudo-allergy (CARPA), which has been associated with nanoparticle administration, is thought of as a hypersensitive “systemic stress response” against infused nanoparticles [237], and can trigger serious reactions such as hypotension, tachycardia, fever, and even death [236, 238]. Therefore, understanding and preventing these infusion reactions is critical to patient health.

*In vivo* models that exhibit similar responses as humans can enable safety testing of LNP formulations and infusion protocols. In particular, pigs are acutely sensitive to nanomaterials and have been a critical model to predict infusion reactions in humans [239]. Among other examples, a porcine CARPA model was utilized to establish safe infusion protocols and measure reactogenicity of PEGylated liposomal prednisolone [240], and methods are also described in U.S. Patent US10246708B2.

One potential strategy to mitigate these responses is through pre-dosing with a prophylactic drug cocktail. For example, patisiran requires patients to be pre-dosed with infusions of dexamethasone, oral acetaminophen/paracetamol, an H2 blocker, and an H1 blocker to mitigate the risk of infusion-related reactions (241). Reduction of LNP dose as well as infusion rate also mitigates this risk [236]. In pigs, administration of complement-inhbiting anti-C5a antibody or soluble CR1, or the cyclooxygenase inhibitor indomethacin mitigated increases in pulmonary arterial pressure caused by liposomes [242].

In an alternative strategy to mitigate CARPA, Wang *et al.* directly conjugated Factor I, which inactivates complement protein C3b, to the surface of liposomes [243]. This modification reduced phagocyte uptake of nanoparticles and mitigated CARPA-associated side effects including increased circulating leukocytes and hematocrit, and cerebral hypoperfusion in mice. In this report, Factor H conjugation was also attempted, but the authors reported that these liposomes tended to be unstable and aggregate.

### Anti-PEG Antibodies

PEGylated lipids are used in LNP formulation to confer steric stability, prevent opsonization, and increase systemic circulation time [69, 244]. However, up to 72% of healthy blood donors tested positive for anti-PEG antibodies, with the prevalence and levels of anti-PEG antibodies increasing with time (compared to historical samples) and patient age [245, 246]. The high prevalence of these antibodies may be due to the extensive use of PEG in consumer products [247]. Repeat injections of LNPs may therefore result in accelerated blood clearance [248, 249]. Interestingly, this does not seem to be the case with patisiran, as repeat administrations have not resulted in significant differences in pharmacokinetics, pharmacodynamics, or efficacy [250, 251]. This may be due to the pre-dose cocktail or the particular composition of patisiran. Nonetheless, the increasing trends of anti-PEG antibody prevalence motivates the development of alternative materials.

Zwitterionic materials comprise both a cationic and anionic charge, such that the net charge is zero. This endows zwitterionic materials with unique properties including strong hydration and mimicry of endogenous lipids, both of which may mitigate protein adsorption and promote immune evasion [252]. Cheng *et al.* tested the pharmacokinetics of native uricase and uricase modified with PEG or encapsulated within zwitterionic carboxybetaine-based nanogels. While multiple injections of native or PEG-modified uricase led to greater rates of clearance, uricase encapsulated within zwitterionic nanogels exhibited essentially identical pharmacokinetic profiles compared to the first injection [253].

### Lessons Learned from Atu027

Atu027 is a formulation of protein kinase 3 (PKN3) siRNA packaged inside liposomes that contain the cationic lipid AtuFECT01, which mediates nucleic acid delivery to endothelial cells [179]. This drug is being investigated for its efficacy against solid tumors, because PKN3 knockdown may prevent metastasis by reducing vascular leakiness and tumor cell migration and tumor lymphangiogenesis and hemangiogenesis. Preclinical studies found that Atu027 reduced metastatic burden in the lung and delayed tumor growth in orthotopic models of prostate and pancreatic cancer [254, 255].

The first clinical trial (NCT00938574) tested the safety, tolerability, and pharmacokinetics/pharmacodynamics of Atu027. Patients with advanced solid tumors (*n* = 34) were administered 10 escalating doses ranging 0.001–0.336 mg/kg without premedication, as a single dose followed by 8 infusions, twice/week, for 4 weeks. Doses up to 0.336 mg/kg were generally tolerated, although one patient experienced dose-limiting toxicity (increased lipase). The prevalence of adverse events did not correlate with dose, and fatigue, increased lipase, and decreased hemoglobin were noted as the most common events. Efficacy wise, 41% of all treated patients exhibited stabilized disease, with one patient exhibited complete regression of a pulmonary lesion [256].

A phase Ib/IIa clinical trial (NCT01808638) evaluated co-administration of Atu027 with the chemotherapeutic gemcitabine; Atu027 was administered either at 0.253 mg/kg once or twice weekly in patients with advanced or metastatic pancreatic adenocarcinoma. Notably, patients administered Atu027 twice/week exhibited greater median progression-free survival (5.3 months) compared to once/week (1.8 months). Greater disease control and reduced numbers of new lesion formation was also observed in patients administered Atu027 twice/week. Grade 3 adverse events (82% and 92%, respectively) were reported for each arm, although it is unclear if these were due to Atu027 or gemcitabine [257].

While these findings are promising and provide a rich perspective on tolerated doses and side effects, the manufacturer Silence Therapeutics has decided to refocus efforts on other platforms due partly to clinical trial costs (Silence Therapeutics, 28 March 2017 Press Release). No information regarding phase III trials has been reported since.

### Alternative Administration Routes

Various injection routes and strategies, compatible with clinically used catheters and access points, can be leveraged to augment nanoparticle concentration in certain tissues and delivery efficiency compared to systemic intravenous routes. Combining the effect of local delivery and vascular targeting on cerebral delivery, Marcos-Contreras *et al.* found that injection of anti-ICAM-1 liposomes via a carotid artery catheter provided a five-fold elevation of accumulation in the brain (tracked by intravital real time microscopy via cranial window) compared to levels obtained by intravenous injection in mice with acute brain inflammation [258]. Another study compared accumulation of radiolabeled anti-PECAM-1 scFv fused with urokinase-type plasminogen activator delivered intra-arterially to the carotid artery, with data suggesting increased cerebral accumulation of the fusion protein by 30% via the arterial route compared to intravenous in mice [259]. Scherpereel *et al.* evaluated local infusion of anti-PECAM-1 via a catheter placed in the right coronary artery of pigs that resulted in a fourfold elevation of cardiac accumulation of anti-PECAM-1 compared to the intravenous route [260]. Therefore, synergizing vascular immunotargeting and catheter placement may enable organ-specific endothelial delivery beyond pulmonary endothelium. Further development of methods to prolong contact with target endothelial tissue and moving towards minimally invasive methods could expedite the translation of these strategies.

## Summary & Outlook

Endothelial cells are the “gateway” to the organs of the body and are significantly involved in cardiovascular disease, diabetes, and cancer. Therefore, modulating endothelial gene expression could stand to impact and improve major chronic diseases. Substantial progress has been made in developing LNPs for nucleic acid delivery to endothelial cells. A major barrier to this field has been hepatic tropism, leading to nucleic acid delivery principally to the liver. Here, we reviewed major strategies to overcome this barrier: while modulation of lipid composition can drive nucleic acid delivery to pulmonary, splenic, and liver cells, these approaches are somewhat limited to these organs. Alternative methods have expanded nucleic acid delivery to other sites: incorporation of cationic lipids or antibodies can enable delivery to the endothelium.

The use of cationic lipids may present translational challenges due to their recognized toxicity [261]. This has motivated the development of ionizable lipids, which are conditionally cationic, as well as the use of antibodies and peptides that bind to endothelial-specific receptors through molecular recognition rather than non-specific electrostatic interactions. Further elucidation of the relationship between the protein corona and tissue tropism [190], coupled with profiling of tissue endothelial cell surface receptors during health and disease, could significantly inform LNP design. Moreover, a recent report highlighted the importance of non-intravenous routes in facilitating nucleic acid delivery to cells of the central nervous system [262], which is challenging to target from the blood space due to the blood–brain barrier. Creative application of alternative routes (e.g., stents, non-intravenous) with materials chemistry and proteomics could further expand the set of targetable tissue vasculature.

Nucleic acid cargos can also offer a degree of spatial (which tissues) and temporal (duration) control of gene modulation. DNA can be programmed with tissue-specific promoters for selective expression; recently mRNA can also be endowed with tissue-specific translation [263]. Moreover, a combination of designed Cas9 mRNA and pre-delivery of a mRNA-inhibiting siRNA to the liver can decrease liver gene editing and augment splenic and lung endothelial editing [264]. The expression duration of mRNA is shorter than that of DNA [101], but both are capable of installing durable genetic edits through encoding of CRISPR/Cas9. Other nucleic acid therapeutics, siRNA, miRNA, and ASOs exhibit transient modulation at the mRNA level; shRNA requires delivery in DNA form and therefore can be controlled in a tissue-specific manner via promoters. Thoughtful combination of LNP chemistry with encapsulated nucleic acids and transcriptional/translational controls can therefore add multiple layers of spatiotemporal control of nucleic acid activity.

Clinical trials and FDA-approved nanoscale therapeutics have informed a range of safe LNP/nucleic acid doses in humans. However, infusion-related reactions are not uncommon and can be life-threatening [236]. While these may be mitigated or obviated with the use of drugs prior to LNP administration (as in the case of patisiran), future efforts could incorporate zwitterionic materials to promote greater immune evasion or directly modify the materials with complement-modulating proteins. Screens of new lipids could equally consider nucleic acid delivery potency and immune activation. The use of porcine models and blood, which are particularly sensitive to nanomaterials, should be considered for screening and testing of LNP formulations. Careful selection of patient populations can also empower statistical analyses of LNP efficacy, although costs may be a major factor as in the case of Atu027 development.

The COVID-19 pandemic has stress-tested the scalability, safety, and efficacy of LNPs, and has engendered greater academic and pharmaceutical investment into this space. The high degree of LNP tailorability and the diversity of nucleic acid cargos position LNPs as a platform with great potential to solve urgent health problems. In particular, we look forward to the next-generation of LNPs capable of nucleic acid delivery to extra-hepatic tissues, particularly to endothelial cells.
